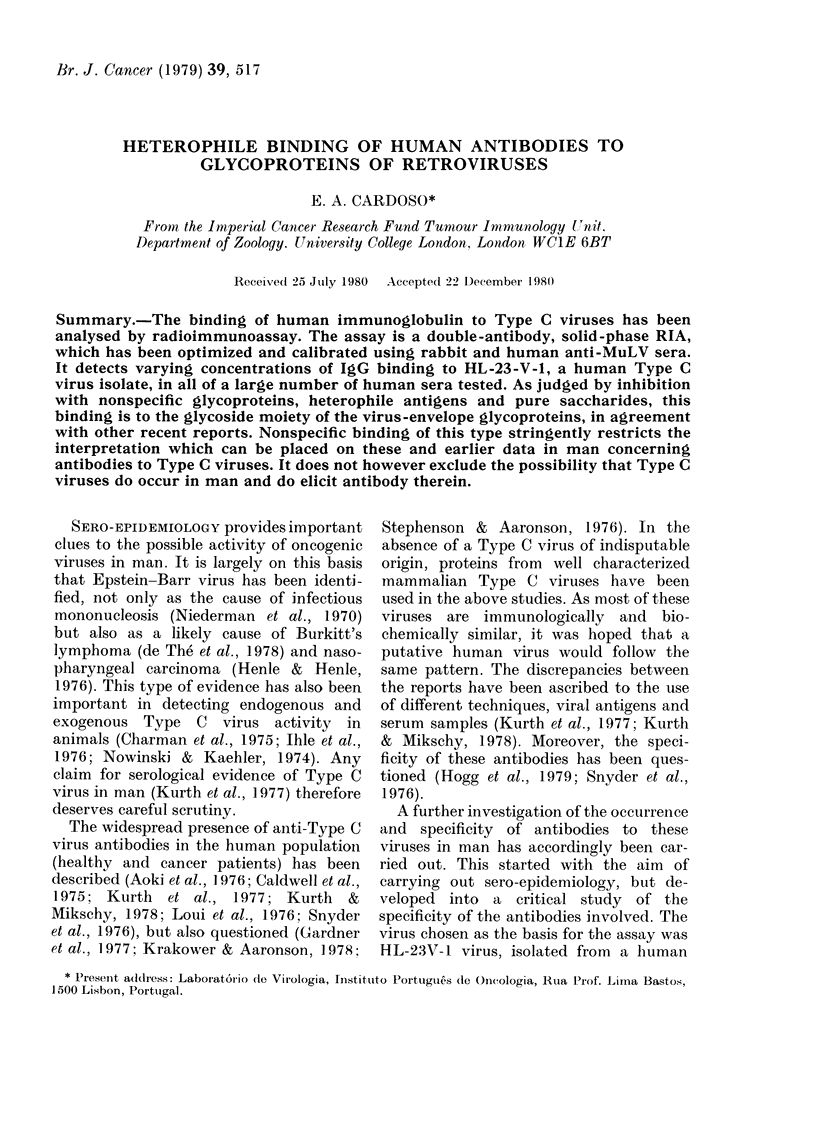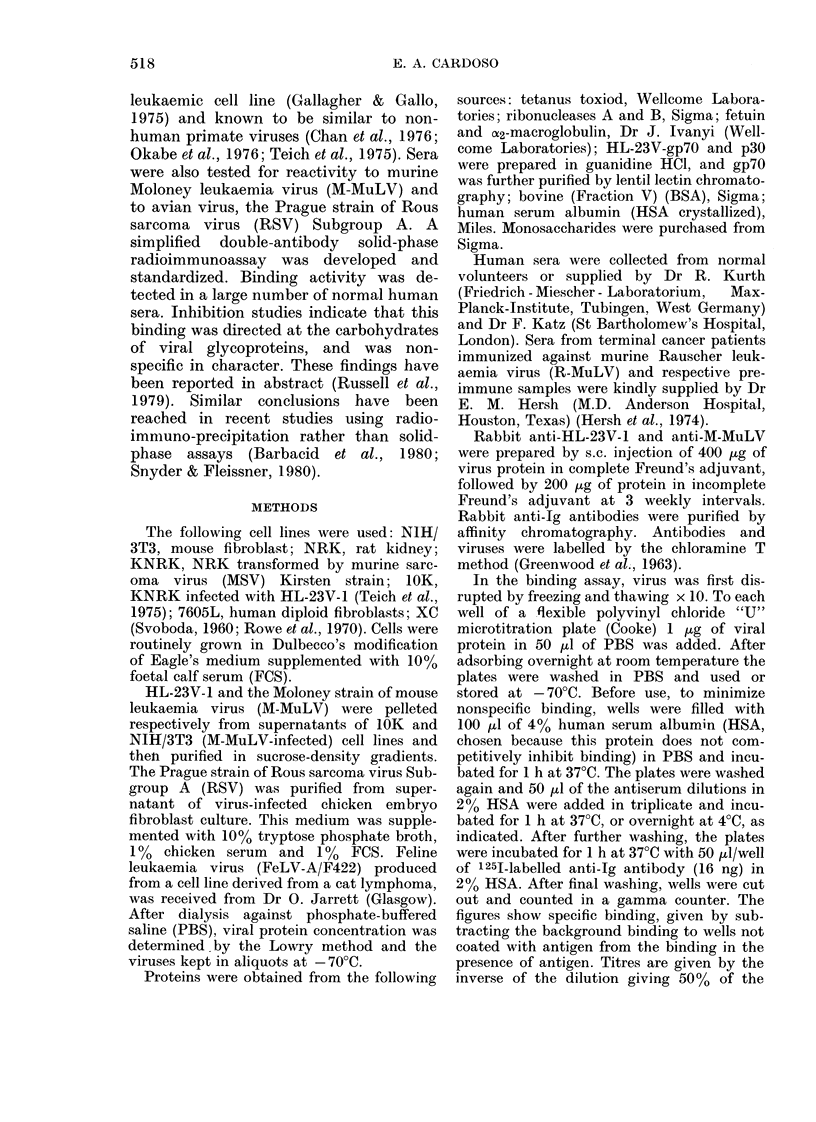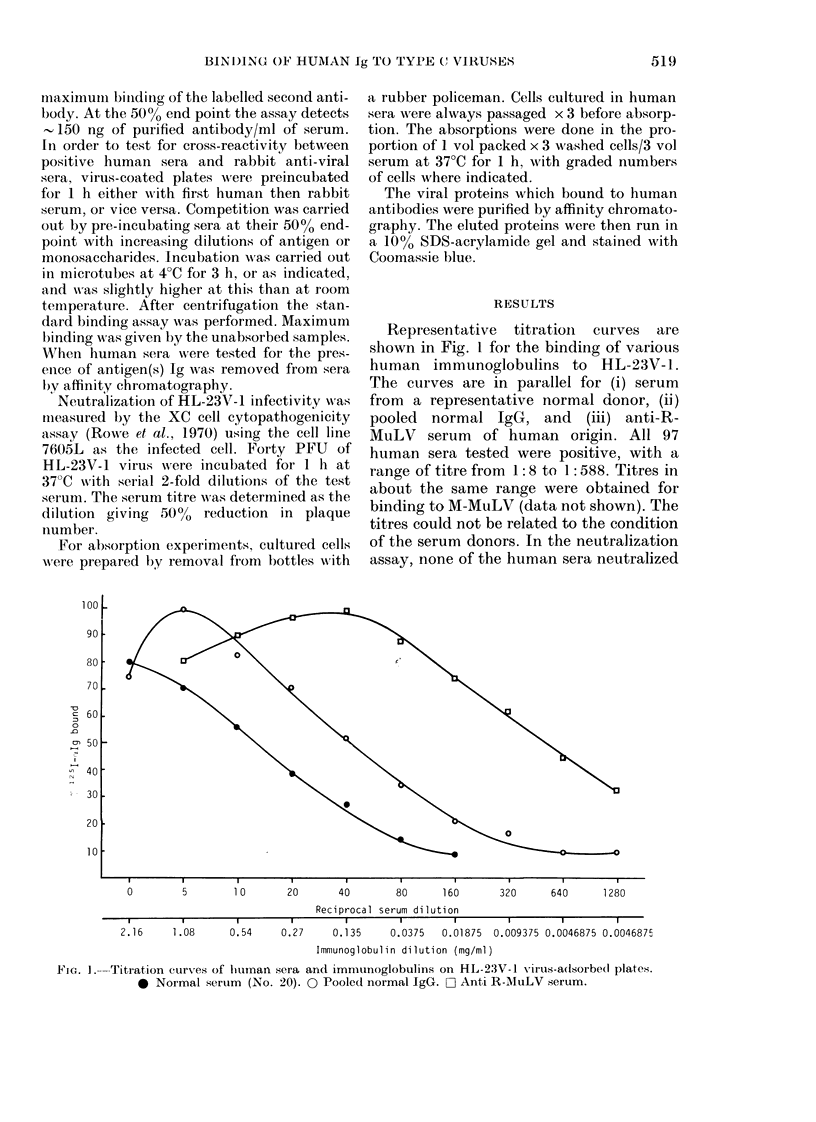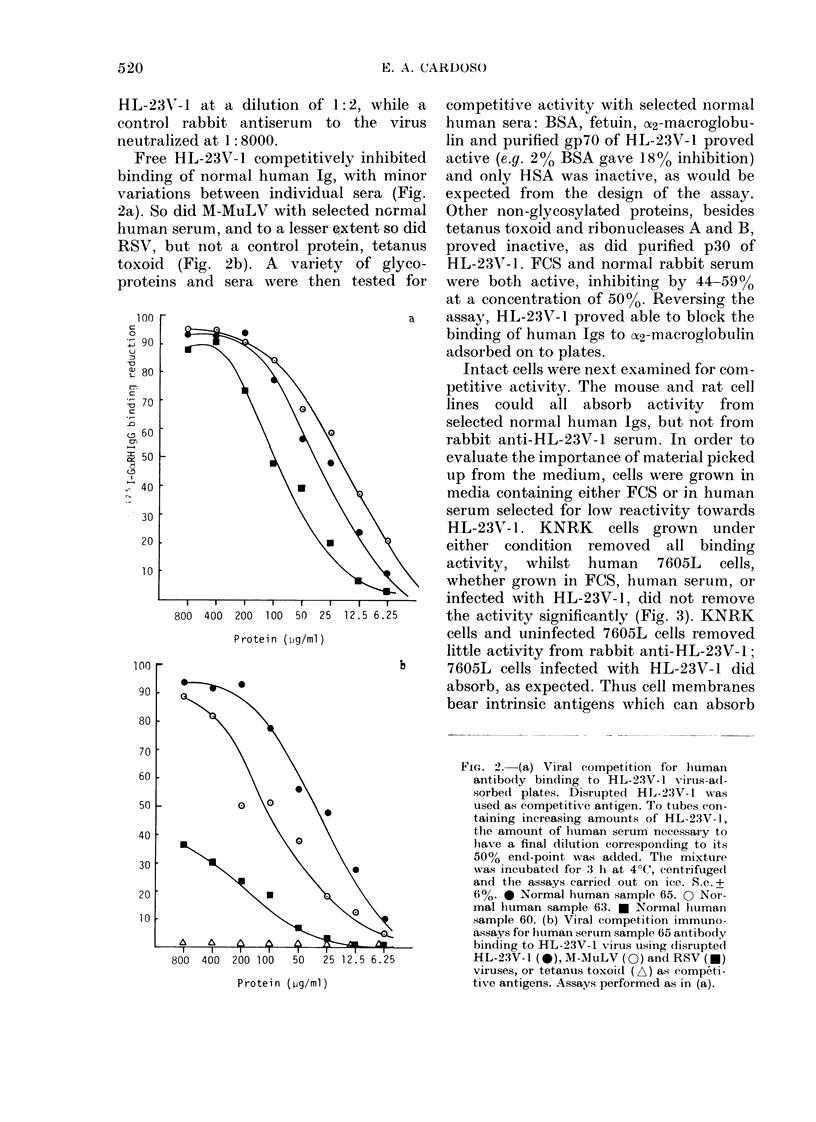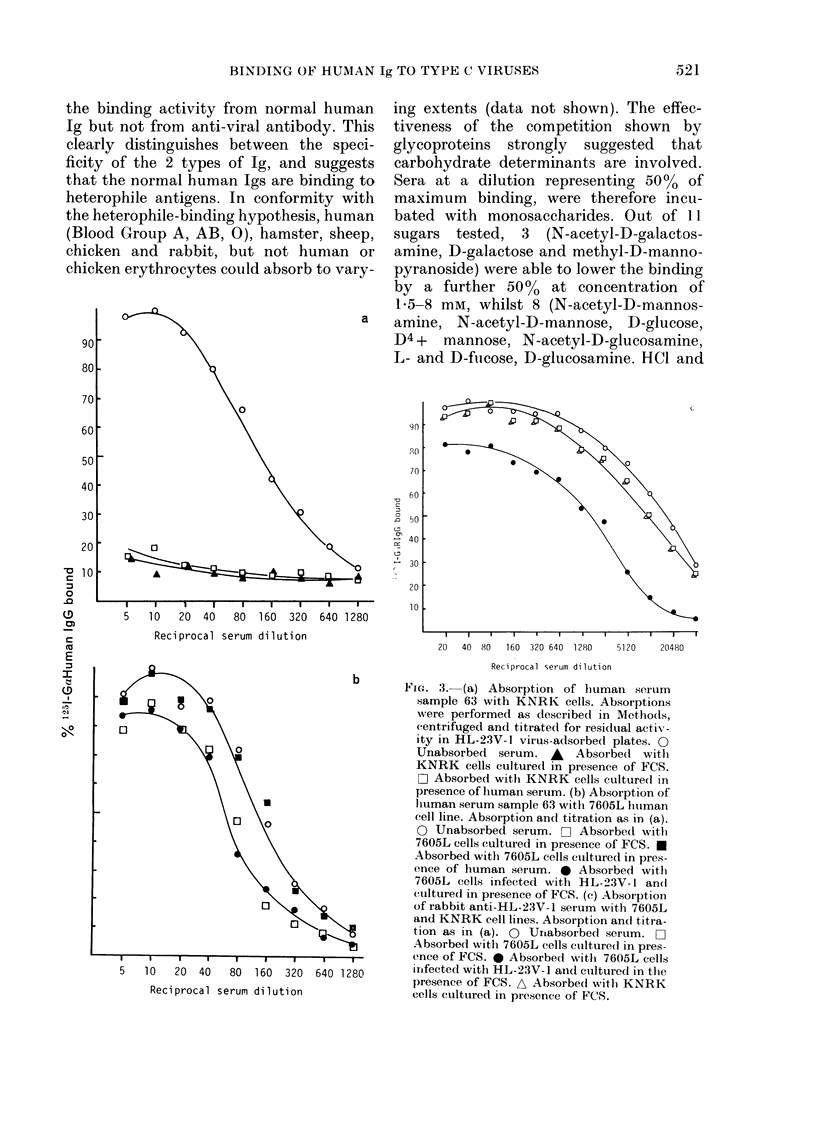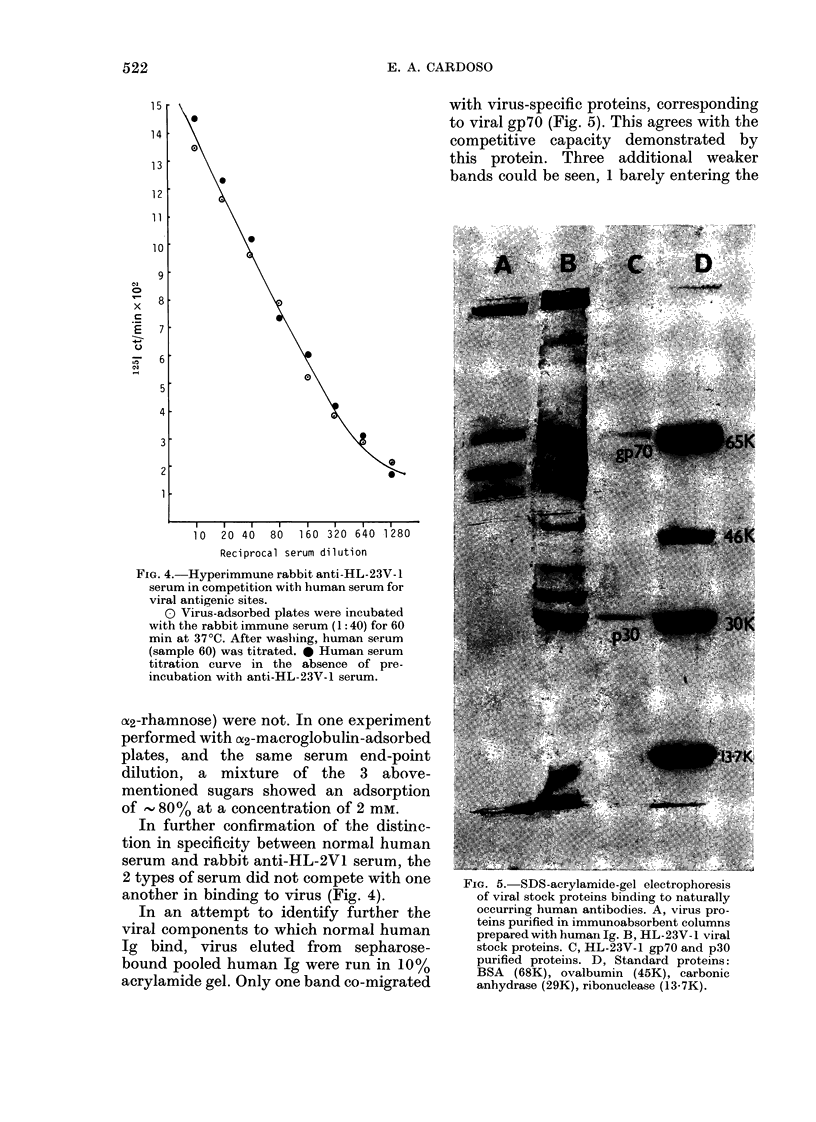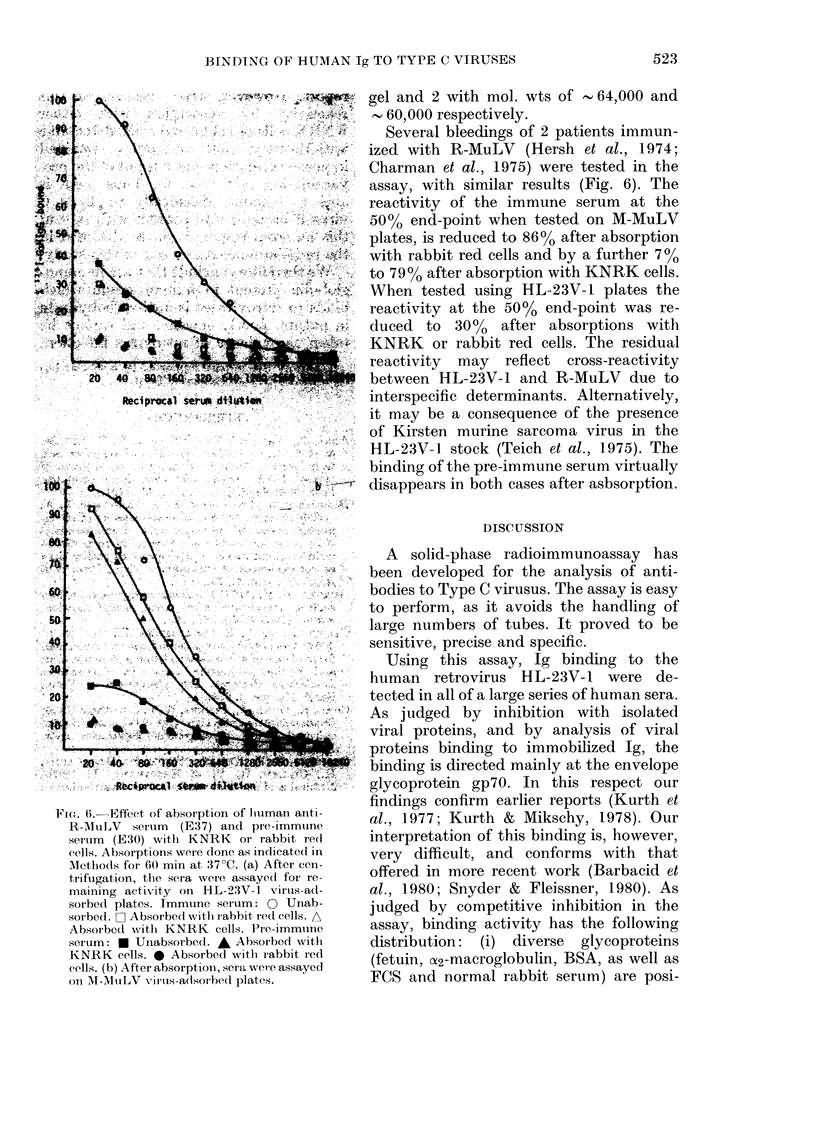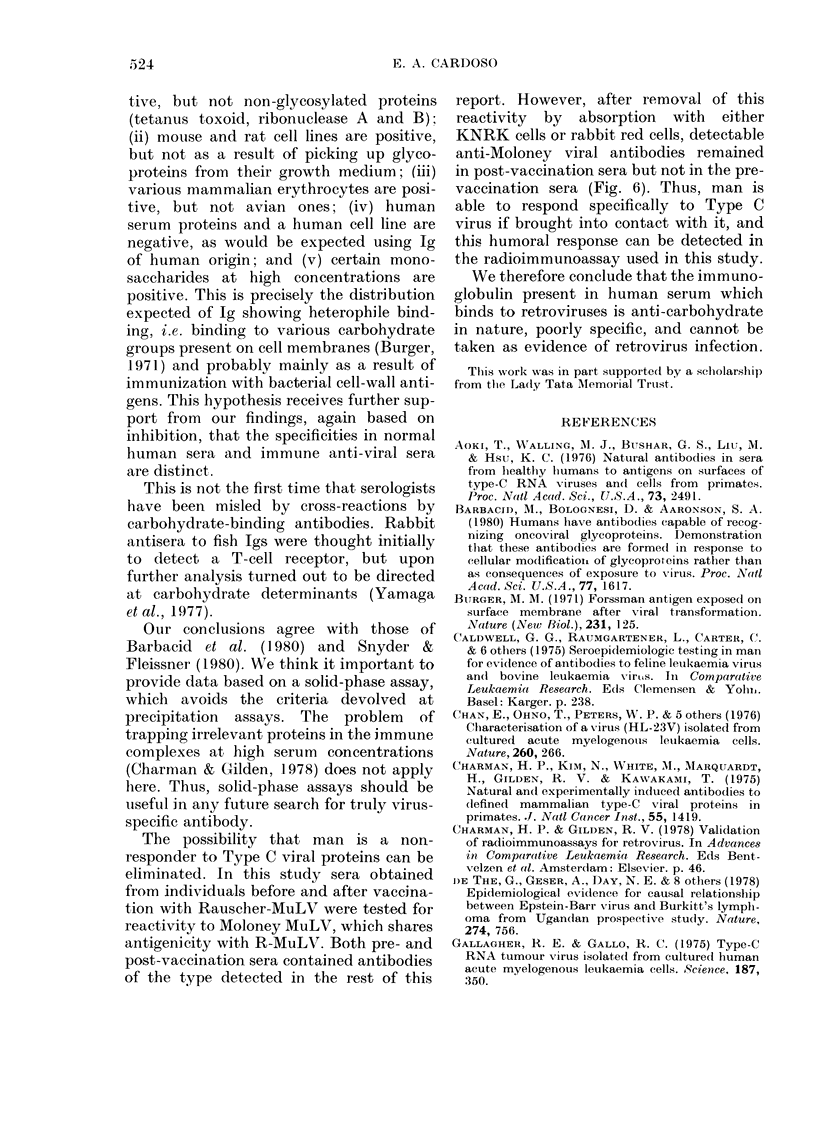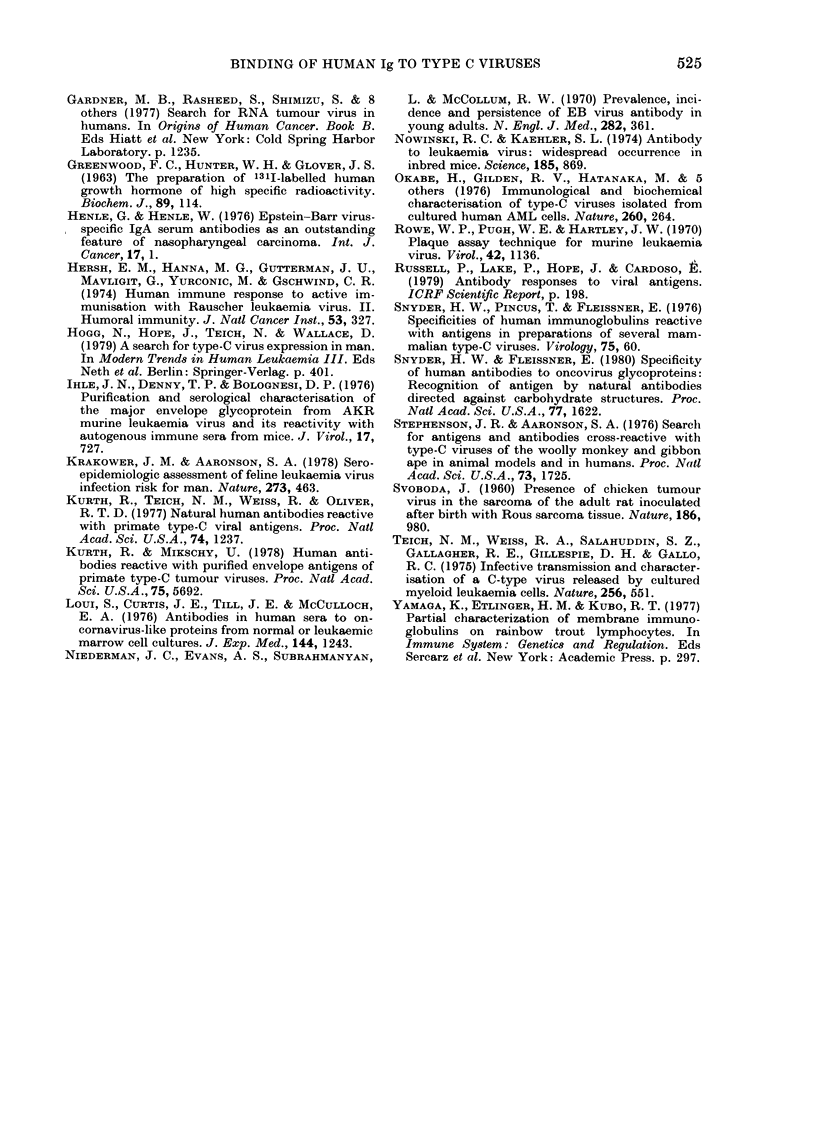# Heterophile binding of human antibodies to glycoproteins of retroviruses.

**DOI:** 10.1038/bjc.1981.74

**Published:** 1981-04

**Authors:** E. A. Cardoso

## Abstract

**Images:**


*Br. J. Cancer* (1979) **39**, 517

### HETEROPHILE BINDING OF HUMAN ANTIBODIES TO GLYCOPROTEINS OF RETROVIRUSES

E. A. CARDOSO*

*From the Imperial Cancer Research Fund Tumour Immunology Unit, Department of Zoology, University College London, London WC1E 6BT*

Received 25 July 1980 Accepted 22 December 1980

**Summary.—The binding of human immunoglobulin to Type C viruses has been analysed by radioimmunoassay. The assay is a double-antibody, solid-phase RIA, which has been optimized and calibrated using rabbit and human anti-MuLV sera. It detects varying concentrations of IgG binding to HL-23-V-1, a human Type C virus isolate, in all of a large number of human sera tested. As judged by inhibition with nonspecific glycoproteins, heterophile antigens and pure saccharides, this binding is to the glycoside moiety of the virus-envelope glycoproteins, in agreement with other recent reports. Nonspecific binding of this type stringently restricts the interpretation which can be placed on these and earlier data in man concerning antibodies to Type C viruses. It does not however exclude the possibility that Type C viruses do occur in man and do elicit antibody therein.**

SERO-EPIDEMIOLOGY provides important clues to the possible activity of oncogenic viruses in man. It is largely on this basis that Epstein–Barr virus has been identified, not only as the cause of infectious mononucleosis (Niederman *et al.*, 1970) but also as a likely cause of Burkitt's lymphoma (de Thé *et al.*, 1978) and nasopharyngeal carcinoma (Henle & Henle, 1976). This type of evidence has also been important in detecting endogenous and exogenous Type C virus activity in animals (Charman *et al.*, 1975; Ihle *et al.*, 1976; Nowinski & Kaehler, 1974). Any claim for serological evidence of Type C virus in man (Kurth *et al.*, 1977) therefore deserves careful scrutiny.

The widespread presence of anti-Type C virus antibodies in the human population (healthy and cancer patients) has been described (Aoki *et al.*, 1976; Caldwell *et al.*, 1975; Kurth *et al.*, 1977; Kurth & Mikschy, 1978; Loui *et al.*, 1976; Snyder *et al.*, 1976), but also questioned (Gardner *et al.*, 1977; Krakower & Aaronson, 1978; Stephenson & Aaronson, 1976). In the absence of a Type C virus of indisputable origin, proteins from well characterized mammalian Type C viruses have been used in the above studies. As most of these viruses are immunologically and biochemically similar, it was hoped that a putative human virus would follow the same pattern. The discrepancies between the reports have been ascribed to the use of different techniques, viral antigens and serum samples (Kurth *et al.*, 1977; Kurth & Mikschy, 1978). Moreover, the specificity of these antibodies has been questioned (Hogg *et al.*, 1979; Snyder *et al.*, 1976).

A further investigation of the occurrence and specificity of antibodies to these viruses in man has accordingly been carried out. This started with the aim of carrying out sero-epidemiology, but developed into a critical study of the specificity of the antibodies involved. The virus chosen as the basis for the assay was HL-23V-1 virus, isolated from a human

* Present address: Laboratório de Virologia, Instituto Português de Oncologia, Rua Prof. Lima Bastos, 1500 Lisbon, Portugal.

leukaemic cell line (Gallagher & Gallo, 1975) and known to be similar to non-human primate viruses (Chan *et al.*, 1976; Okabe *et al.*, 1976; Teich *et al.*, 1975). Sera were also tested for reactivity to murine Moloney leukaemia virus (M-MuLV) and to avian virus, the Prague strain of Rous sarcoma virus (RSV) Subgroup A. A simplified double-antibody solid-phase radioimmunoassay was developed and standardized. Binding activity was detected in a large number of normal human sera. Inhibition studies indicate that this binding was directed at the carbohydrates of viral glycoproteins, and was non-specific in character. These findings have been reported in abstract (Russell *et al.*, 1979). Similar conclusions have been reached in recent studies using radioimmuno-precipitation rather than solid-phase assays (Barbacid *et al.*, 1980; Snyder & Fleissner, 1980).

### METHODS

The following cell lines were used: NIH/3T3, mouse fibroblast; NRK, rat kidney; KNRK, NRK transformed by murine sarcoma virus (MSV) Kirsten strain; 10K, KNRK infected with HL-23V-1 (Teich *et al.*, 1975); 7605L, human diploid fibroblasts; XC (Svoboda, 1960; Rowe *et al.*, 1970). Cells were routinely grown in Dulbecco's modification of Eagle's medium supplemented with 10% foetal calf serum (FCS).

HL-23V-1 and the Moloney strain of mouse leukaemia virus (M-MuLV) were pelleted respectively from supernatants of 10K and NIH/3T3 (M-MuLV-infected) cell lines and then purified in sucrose-density gradients. The Prague strain of Rous sarcoma virus Subgroup A (RSV) was purified from supernatant of virus-infected chicken embryo fibroblast culture. This medium was supplemented with 10% tryptose phosphate broth, 1% chicken serum and 1% FCS. Feline leukaemia virus (FeLV-A/F422) produced from a cell line derived from a cat lymphoma, was received from Dr O. Jarrett (Glasgow). After dialysis against phosphate-buffered saline (PBS), viral protein concentration was determined by the Lowry method and the viruses kept in aliquots at $-70$°C.

Proteins were obtained from the following sources: tetanus toxiod, Wellcome Laboratories; ribonucleases A and B, Sigma; fetuin and $\alpha_2$-macroglobulin, Dr J. Ivanyi (Wellcome Laboratories); HL-23V-gp70 and p30 were prepared in guanidine HCl, and gp70 was further purified by lentil lectin chromatography; bovine (Fraction V) (BSA), Sigma; human serum albumin (HSA crystallized), Miles. Monosaccharides were purchased from Sigma.

Human sera were collected from normal volunteers or supplied by Dr R. Kurth (Friedrich - Miescher - Laboratorium, Max-Planck-Institute, Tubingen, West Germany) and Dr F. Katz (St Bartholomew's Hospital, London). Sera from terminal cancer patients immunized against murine Rauscher leukaemia virus (R-MuLV) and respective pre-immune samples were kindly supplied by Dr E. M. Hersh (M.D. Anderson Hospital, Houston, Texas) (Hersh *et al.*, 1974).

Rabbit anti-HL-23V-1 and anti-M-MuLV were prepared by s.c. injection of 400 $\mu$g of virus protein in complete Freund's adjuvant, followed by 200 $\mu$g of protein in incomplete Freund's adjuvant at 3 weekly intervals. Rabbit anti-Ig antibodies were purified by affinity chromatography. Antibodies and viruses were labelled by the chloramine T method (Greenwood *et al.*, 1963).

In the binding assay, virus was first disrupted by freezing and thawing $\times 10$. To each well of a flexible polyvinyl chloride "U" microtitration plate (Cooke) 1 $\mu$g of viral protein in 50 $\mu$l of PBS was added. After adsorbing overnight at room temperature the plates were washed in PBS and used or stored at $-70$°C. Before use, to minimize nonspecific binding, wells were filled with 100 $\mu$l of 4% human serum albumin (HSA, chosen because this protein does not competitively inhibit binding) in PBS and incubated for 1 h at 37°C. The plates were washed again and 50 $\mu$l of the antiserum dilutions in 2% HSA were added in triplicate and incubated for 1 h at 37°C, or overnight at 4°C, as indicated. After further washing, the plates were incubated for 1 h at 37°C with 50 $\mu$l/well of $^{125}$I-labelled anti-Ig antibody (16 ng) in 2% HSA. After final washing, wells were cut out and counted in a gamma counter. The figures show specific binding, given by subtracting the background binding to wells not coated with antigen from the binding in the presence of antigen. Titres are given by the inverse of the dilution giving 50% of the maximum binding of the labelled second antibody. At the 50% end point the assay detects ~150 ng of purified antibody/ml of serum. In order to test for cross-reactivity between positive human sera and rabbit anti-viral sera, virus-coated plates were preincubated for 1 h either with first human then rabbit serum, or vice versa. Competition was carried out by pre-incubating sera at their 50% end-point with increasing dilutions of antigen or monosaccharides. Incubation was carried out in microtubes at 4°C for 3 h, or as indicated, and was slightly higher at this than at room temperature. After centrifugation the standard binding assay was performed. Maximum binding was given by the unabsorbed samples. When human sera were tested for the presence of antigen(s) Ig was removed from sera by affinity chromatography.

Neutralization of HL-23V-1 infectivity was measured by the XC cell cytopathogenicity assay (Rowe *et al.*, 1970) using the cell line 7605L as the infected cell. Forty PFU of HL-23V-1 virus were incubated for 1 h at 37°C with serial 2-fold dilutions of the test serum. The serum titre was determined as the dilution giving 50% reduction in plaque number.

For absorption experiments, cultured cells were prepared by removal from bottles with a rubber policeman. Cells cultured in human sera were always passaged ×3 before absorption. The absorptions were done in the proportion of 1 vol packed ×3 washed cells/3 vol serum at 37°C for 1 h, with graded numbers of cells where indicated.

The viral proteins which bound to human antibodies were purified by affinity chromatography. The eluted proteins were then run in a 10% SDS-acrylamide gel and stained with Coomassie blue.

### RESULTS

Representative titration curves are shown in Fig. 1 for the binding of various human immunoglobulins to HL-23V-1. The curves are in parallel for (i) serum from a representative normal donor, (ii) pooled normal IgG, and (iii) anti-R-MuLV serum of human origin. All 97 human sera tested were positive, with a range of titre from 1:8 to 1:588. Titres in about the same range were obtained for binding to M-MuLV (data not shown). The titres could not be related to the condition of the serum donors. In the neutralization assay, none of the human sera neutralized

FIG. 1.—Titration curves of human sera and immunoglobulins on HL-23V-1 virus-adsorbed plates. ● Normal serum (No. 20). ○ Pooled normal IgG. □ Anti R-MuLV serum.

HL-23V-1 at a dilution of 1:2, while a control rabbit antiserum to the virus neutralized at 1:8000.

Free HL-23V-1 competitively inhibited binding of normal human Ig, with minor variations between individual sera (Fig. 2a). So did M-MuLV with selected normal human serum, and to a lesser extent so did RSV, but not a control protein, tetanus toxoid (Fig. 2b). A variety of glycoproteins and sera were then tested for competitive activity with selected normal human sera: BSA, fetuin, $\alpha_2$-macroglobulin and purified gp70 of HL-23V-1 proved active (*e.g.* 2% BSA gave 18% inhibition) and only HSA was inactive, as would be expected from the design of the assay. Other non-glycosylated proteins, besides tetanus toxoid and ribonucleases A and B, proved inactive, as did purified p30 of HL-23V-1. FCS and normal rabbit serum were both active, inhibiting by 44–59% at a concentration of 50%. Reversing the assay, HL-23V-1 proved able to block the binding of human Igs to $\alpha_2$-macroglobulin adsorbed on to plates.

Intact cells were next examined for competitive activity. The mouse and rat cell lines could all absorb activity from selected normal human Igs, but not from rabbit anti-HL-23V-1 serum. In order to evaluate the importance of material picked up from the medium, cells were grown in media containing either FCS or in human serum selected for low reactivity towards HL-23V-1. KNRK cells grown under either condition removed all binding activity, whilst human 7605L cells, whether grown in FCS, human serum, or infected with HL-23V-1, did not remove the activity significantly (Fig. 3). KNRK cells and uninfected 7605L cells removed little activity from rabbit anti-HL-23V-1; 7605L cells infected with HL-23V-1 did absorb, as expected. Thus cell membranes bear intrinsic antigens which can absorb

FIG. 2.—(a) Viral competition for human antibody binding to HL-23V-1 virus-adsorbed plates. Disrupted HL-23V-1 was used as competitive antigen. To tubes containing increasing amounts of HL-23V-1, the amount of human serum necessary to have a final dilution corresponding to its 50% end-point was added. The mixture was incubated for 3 h at 4°C, centrifuged and the assays carried out on ice. S.e. ± 6%. ● Normal human sample 65. ○ Normal human sample 63. ■ Normal human sample 60. (b) Viral competition immunoassays for human serum sample 65 antibody binding to HL-23V-1 virus using disrupted HL-23V-1 (●), M-MuLV (○) and RSV (■) viruses, or tetanus toxoid (△) as competitive antigens. Assays performed as in (a).

the binding activity from normal human Ig but not from anti-viral antibody. This clearly distinguishes between the specificity of the 2 types of Ig, and suggests that the normal human Igs are binding to heterophile antigens. In conformity with the heterophile-binding hypothesis, human (Blood Group A, AB, O), hamster, sheep, chicken and rabbit, but not human or chicken erythrocytes could absorb to varying extents (data not shown). The effectiveness of the competition shown by glycoproteins strongly suggested that carbohydrate determinants are involved. Sera at a dilution representing 50% of maximum binding, were therefore incubated with monosaccharides. Out of 11 sugars tested, 3 (N-acetyl-D-galactosamine, D-galactose and methyl-D-mannopyranoside) were able to lower the binding by a further 50% at concentration of 1·5–8 mM, whilst 8 (N-acetyl-D-mannosamine, N-acetyl-D-mannose, D-glucose, D4+ mannose, N-acetyl-D-glucosamine, L- and D-fucose, D-glucosamine. HCl and

FIG. 3.—(a) Absorption of human serum sample 63 with KNRK cells. Absorptions were performed as described in Methods, centrifuged and titrated for residual activity in HL-23V-1 virus-adsorbed plates. ○ Unabsorbed serum. ▲ Absorbed with KNRK cells cultured in presence of FCS. □ Absorbed with KNRK cells cultured in presence of human serum. (b) Absorption of human serum sample 63 with 7605L human cell line. Absorption and titration as in (a). ○ Unabsorbed serum. □ Absorbed with 7605L cells cultured in presence of FCS. ■ Absorbed with 7605L cells cultured in presence of human serum. ● Absorbed with 7605L cells infected with HL-23V-1 and cultured in presence of FCS. (c) Absorption of rabbit anti-HL-23V-1 serum with 7605L and KNRK cell lines. Absorption and titration as in (a). ○ Unabsorbed serum. □ Absorbed with 7605L cells cultured in presence of FCS. ● Absorbed with 7605L cells infected with HL-23V-1 and cultured in the presence of FCS. △ Absorbed with KNRK cells cultured in presence of FCS.

FIG. 4.—Hyperimmune rabbit anti-HL-23V-1 serum in competition with human serum for viral antigenic sites.

⊙ Virus-adsorbed plates were incubated with the rabbit immune serum (1:40) for 60 min at 37°C. After washing, human serum (sample 60) was titrated. ● Human serum titration curve in the absence of pre-incubation with anti-HL-23V-1 serum.

$\alpha_2$-rhamnose) were not. In one experiment performed with $\alpha_2$-macroglobulin-adsorbed plates, and the same serum end-point dilution, a mixture of the 3 above-mentioned sugars showed an adsorption of ~80% at a concentration of 2 mM.

In further confirmation of the distinction in specificity between normal human serum and rabbit anti-HL-2V1 serum, the 2 types of serum did not compete with one another in binding to virus (Fig. 4).

In an attempt to identify further the viral components to which normal human Ig bind, virus eluted from sepharose-bound pooled human Ig were run in 10% acrylamide gel. Only one band co-migrated with virus-specific proteins, corresponding to viral gp70 (Fig. 5). This agrees with the competitive capacity demonstrated by this protein. Three additional weaker bands could be seen, 1 barely entering the

FIG. 5.—SDS-acrylamide-gel electrophoresis of viral stock proteins binding to naturally occurring human antibodies. A, virus proteins purified in immunoabsorbent columns prepared with human Ig. B, HL-23V-1 viral stock proteins. C, HL-23V-1 gp70 and p30 purified proteins. D, Standard proteins: BSA (68K), ovalbumin (45K), carbonic anhydrase (29K), ribonuclease (13·7K).

FIG. 6.—Effect of absorption of human anti-R-MuLV serum (E37) and pre-immune serum (E30) with KNRK or rabbit red cells. Absorptions were done as indicated in Methods for 60 min at 37°C. (a) After centrifugation, the sera were assayed for remaining activity on HL-23V-1 virus-adsorbed plates. Immune serum: ○ Unabsorbed. □ Absorbed with rabbit red cells. △ Absorbed with KNRK cells. Pre-immune serum: ■ Unabsorbed. ▲ Absorbed with KNRK cells. ● Absorbed with rabbit red cells. (b) After absorption, sera were assayed on M-MuLV virus-adsorbed plates.

gel and 2 with mol. wts of ~64,000 and ~60,000 respectively.

Several bleedings of 2 patients immunized with R-MuLV (Hersh *et al.*, 1974; Charman *et al.*, 1975) were tested in the assay, with similar results (Fig. 6). The reactivity of the immune serum at the 50% end-point when tested on M-MuLV plates, is reduced to 86% after absorption with rabbit red cells and by a further 7% to 79% after absorption with KNRK cells. When tested using HL-23V-1 plates the reactivity at the 50% end-point was reduced to 30% after absorptions with KNRK or rabbit red cells. The residual reactivity may reflect cross-reactivity between HL-23V-1 and R-MuLV due to interspecific determinants. Alternatively, it may be a consequence of the presence of Kirsten murine sarcoma virus in the HL-23V-1 stock (Teich *et al.*, 1975). The binding of the pre-immune serum virtually disappears in both cases after asbsorption.

### DISCUSSION

A solid-phase radioimmunoassay has been developed for the analysis of antibodies to Type C virusus. The assay is easy to perform, as it avoids the handling of large numbers of tubes. It proved to be sensitive, precise and specific.

Using this assay, Ig binding to the human retrovirus HL-23V-1 were detected in all of a large series of human sera. As judged by inhibition with isolated viral proteins, and by analysis of viral proteins binding to immobilized Ig, the binding is directed mainly at the envelope glycoprotein gp70. In this respect our findings confirm earlier reports (Kurth *et al.*, 1977; Kurth & Mikschy, 1978). Our interpretation of this binding is, however, very difficult, and conforms with that offered in more recent work (Barbacid *et al.*, 1980; Snyder & Fleissner, 1980). As judged by competitive inhibition in the assay, binding activity has the following distribution: (i) diverse glycoproteins (fetuin, $\alpha_2$-macroglobulin, BSA, as well as FCS and normal rabbit serum) are positive, but not non-glycosylated proteins (tetanus toxoid, ribonuclease A and B); (ii) mouse and rat cell lines are positive, but not as a result of picking up glycoproteins from their growth medium; (iii) various mammalian erythrocytes are positive, but not avian ones; (iv) human serum proteins and a human cell line are negative, as would be expected using Ig of human origin; and (v) certain monosaccharides at high concentrations are positive. This is precisely the distribution expected of Ig showing heterophile binding, *i.e.* binding to various carbohydrate groups present on cell membranes (Burger, 1971) and probably mainly as a result of immunization with bacterial cell-wall antigens. This hypothesis receives further support from our findings, again based on inhibition, that the specificities in normal human sera and immune anti-viral sera are distinct.

This is not the first time that serologists have been misled by cross-reactions by carbohydrate-binding antibodies. Rabbit antisera to fish Igs were thought initially to detect a T-cell receptor, but upon further analysis turned out to be directed at carbohydrate determinants (Yamaga *et al.*, 1977).

Our conclusions agree with those of Barbacid *et al.* (1980) and Snyder & Fleissner (1980). We think it important to provide data based on a solid-phase assay, which avoids the criteria devolved at precipitation assays. The problem of trapping irrelevant proteins in the immune complexes at high serum concentrations (Charman & Gilden, 1978) does not apply here. Thus, solid-phase assays should be useful in any future search for truly virus-specific antibody.

The possibility that man is a non-responder to Type C viral proteins can be eliminated. In this study sera obtained from individuals before and after vaccination with Rauscher-MuLV were tested for reactivity to Moloney MuLV, which shares antigenicity with R-MuLV. Both pre- and post-vaccination sera contained antibodies of the type detected in the rest of this report. However, after removal of this reactivity by absorption with either KNRK cells or rabbit red cells, detectable anti-Moloney viral antibodies remained in post-vaccination sera but not in the pre-vaccination sera (Fig. 6). Thus, man is able to respond specifically to Type C virus if brought into contact with it, and this humoral response can be detected in the radioimmunoassay used in this study.

We therefore conclude that the immunoglobulin present in human serum which binds to retroviruses is anti-carbohydrate in nature, poorly specific, and cannot be taken as evidence of retrovirus infection.

This work was in part supported by a scholarship from the Lady Tata Memorial Trust.

### REFERENCES

AOKI, T., WALLING, M. J., BUSHAR, G. S., LIU, M. & HSU, K. C. (1976) Natural antibodies in sera from healthy humans to antigens on surfaces of type-C RNA viruses and cells from primates. *Proc. Natl Acad. Sci., U.S.A.*, **73**, 2491.

BARBACID, M., BOLOGNESI, D. & AARONSON, S. A. (1980) Humans have antibodies capable of recognizing oncoviral glycoproteins. Demonstration that these antibodies are formed in response to cellular modification of glycoproteins rather than as consequences of exposure to virus. *Proc. Natl Acad. Sci. U.S.A.*, **77**, 1617.

BURGER, M. M. (1971) Forssman antigen exposed on surface membrane after viral transformation. *Nature (New Biol.)*, **231**, 125.

CALDWELL, G. G., RAUMGARTENER, L., CARTER, C. & 6 others (1975) Seroepidemiologic testing in man for evidence of antibodies to feline leukaemia virus and bovine leukaemia virus. In *Comparative Leukaemia Research*. Eds Clemensen & Yohn. Basel: Karger. p. 238.

CHAN, E., OHNO, T., PETERS, W. P. & 5 others (1976) Characterisation of a virus (HL-23V) isolated from cultured acute myelogenous leukaemia cells. *Nature*, **260**, 266.

CHARMAN, H. P., KIM, N., WHITE, M., MARQUARDT, H., GILDEN, R. V. & KAWAKAMI, T. (1975) Natural and experimentally induced antibodies to defined mammalian type-C viral proteins in primates. *J. Natl Cancer Inst.*, **55**, 1419.

CHARMAN, H. P. & GILDEN, R. V. (1978) Validation of radioimmunoassays for retrovirus. In *Advances in Comparative Leukaemia Research*. Eds Bentvelzen *et al.* Amsterdam: Elsevier. p. 46.

DE THE, G., GESER, A., DAY, N. E. & 8 others (1978) Epidemiological evidence for causal relationship between Epstein-Barr virus and Burkitt's lymphoma from Ugandan prospective study. *Nature*, **274**, 756.

GALLAGHER, R. E. & GALLO, R. C. (1975) Type-C RNA tumour virus isolated from cultured human acute myelogenous leukaemia cells. *Science*, **187**, 350.

GARDNER, M. B., RASHEED, S., SHIMIZU, S. & 8 others (1977) Search for RNA tumour virus in humans. In *Origins of Human Cancer. Book B.* Eds Hiatt *et al.* New York: Cold Spring Harbor Laboratory. p. 1235.

GREENWOOD, F. C., HUNTER, W. H. & GLOVER, J. S. (1963) The preparation of $^{131}$I-labelled human growth hormone of high specific radioactivity. *Biochem. J.*, **89**, 114.

HENLE, G. & HENLE, W. (1976) Epstein–Barr virus-specific IgA serum antibodies as an outstanding feature of nasopharyngeal carcinoma. *Int. J. Cancer*, **17**, 1.

HERSH, E. M., HANNA, M. G., GUTTERMAN, J. U., MAVLIGIT, G., YURCONIC, M. & GSCHWIND, C. R. (1974) Human immune response to active immunisation with Rauscher leukaemia virus. II. Humoral immunity. *J. Natl Cancer Inst.*, **53**, 327.

HOGG, N., HOPE, J., TEICH, N. & WALLACE, D. (1979) A search for type-C virus expression in man. In *Modern Trends in Human Leukaemia III.* Eds Neth *et al.* Berlin: Springer-Verlag. p. 401.

IHLE, J. N., DENNY, T. P. & BOLOGNESI, D. P. (1976) Purification and serological characterisation of the major envelope glycoprotein from AKR murine leukaemia virus and its reactivity with autogenous immune sera from mice. *J. Virol.*, **17**, 727.

KRAKOWER, J. M. & AARONSON, S. A. (1978) Sero-epidemiologic assessment of feline leukaemia virus infection risk for man. *Nature*, **273**, 463.

KURTH, R., TEICH, N. M., WEISS, R. & OLIVER, R. T. D. (1977) Natural human antibodies reactive with primate type-C viral antigens. *Proc. Natl Acad. Sci. U.S.A.*, **74**, 1237.

KURTH, R. & MIKSCHY, U. (1978) Human antibodies reactive with purified envelope antigens of primate type-C tumour viruses. *Proc. Natl Acad. Sci. U.S.A.*, **75**, 5692.

LOUI, S., CURTIS, J. E., TILL, J. E. & MCCULLOCH, E. A. (1976) Antibodies in human sera to oncornavirus-like proteins from normal or leukaemic marrow cell cultures. *J. Exp. Med.*, **144**, 1243.

NIEDERMAN, J. C., EVANS, A. S., SUBRAHMANYAN, L. & MCCOLLUM, R. W. (1970) Prevalence, incidence and persistence of EB virus antibody in young adults. *N. Engl. J. Med.*, **282**, 361.

NOWINSKI, R. C. & KAEHLER, S. L. (1974) Antibody to leukaemia virus: widespread occurrence in inbred mice. *Science*, **185**, 869.

OKABE, H., GILDEN, R. V., HATANAKA, M. & 5 others (1976) Immunological and biochemical characterisation of type-C viruses isolated from cultured human AML cells. *Nature*, **260**, 264.

ROWE, W. P., PUGH, W. E. & HARTLEY, J. W. (1970) Plaque assay technique for murine leukaemia virus. *Virol.*, **42**, 1136.

RUSSELL, P., LAKE, P., HOPE, J. & CARDOSO, E. (1979) Antibody responses to viral antigens. *ICRF Scientific Report*, p. 198.

SNYDER, H. W., PINCUS, T. & FLEISSNER, E. (1976) Specificities of human immunoglobulins reactive with antigens in preparations of several mammalian type-C viruses. *Virology*, **75**, 60.

SNYDER, H. W. & FLEISSNER, E. (1980) Specificity of human antibodies to oncovirus glycoproteins: Recognition of antigen by natural antibodies directed against carbohydrate structures. *Proc. Natl Acad. Sci. U.S.A.*, **77**, 1622.

STEPHENSON, J. R. & AARONSON, S. A. (1976) Search for antigens and antibodies cross-reactive with type-C viruses of the woolly monkey and gibbon ape in animal models and in humans. *Proc. Natl Acad. Sci. U.S.A.*, **73**, 1725.

SVOBODA, J. (1960) Presence of chicken tumour virus in the sarcoma of the adult rat inoculated after birth with Rous sarcoma tissue. *Nature*, **186**, 980.

TEICH, N. M., WEISS, R. A., SALAHUDDIN, S. Z., GALLAGHER, R. E., GILLESPIE, D. H. & GALLO, R. C. (1975) Infective transmission and characterisation of a C-type virus released by cultured myeloid leukaemia cells. *Nature*, **256**, 551.

YAMAGA, K., ETLINGER, H. M. & KUBO, R. T. (1977) Partial characterization of membrane immunoglobulins on rainbow trout lymphocytes. In *Immune System: Genetics and Regulation.* Eds Sercarz *et al.* New York: Academic Press. p. 297.